# Polymer-Decorated Cellulose Nanocrystals as Environmentally Friendly Additives for Olefin-Based Drilling Fluids

**DOI:** 10.3390/ijms22010352

**Published:** 2020-12-31

**Authors:** José Aurélio Pinheiro, Nívia do Nascimento Marques, Marcos Antônio Villetti, Rosangela de Carvalho Balaban

**Affiliations:** 1 Laboratório de Pesquisa em Petróleo, Universidade Federal do Rio Grande do Norte, Natal, RN 59078-970, Brazil; joseaureliopinheiro@gmail.com (J.A.P.); nivianmarques@hotmail.com (N.d.N.M.); 2 Departamento de Física, Centro de Ciências Naturais e Exatas, Universidade Federal de Santa Maria, Santa Maria, RS 97105-900, Brazil; mvilletti@hotmail.com

**Keywords:** CNC, PNIPAM, grafting from, water-in-oil emulsion fluid, filtrate reducer

## Abstract

In this study, we intended to evaluate the performance of olefin-based drilling fluids after addition of cellulose nanocrystal (CNC) derivatives. For this purpose, firstly, cellulose nanocrystals, produced from sulfuric acid hydrolysis of cotton fibers, were functionalized with poly(*N*-isopropylacrylamide) (PNIPAM) chains via free radicals. The samples were then characterized via Fourier transform infrared spectroscopy (FTIR), nuclear magnetic resonance (NMR), X-ray diffraction (XRD), thermogravimetric analysis (TGA), confocal microscopy, dynamic light scattering (DLS), and zeta potential measurements in water. The FTIR and NMR spectra exhibited the characteristic signals of CNC and PNIPAM groups, indicating successful grafting. As expected, X-ray diffractograms showed that the crystallinity of CNCs reduces after chemical modification. TGA revealed that the surface-functionalized CNCs present higher thermal stability than pure CNCs. The confocal microscopy, zeta potential, and DLS results were consistent with the behavior of cellulose nanocrystals decorated by a shell of PNIPAM chains. The fluids with a small amount of modified CNCs presented a much lower volume of filtrate after high-temperature and high-pressure (HTHP) filtration tests than the corresponding standard fluid, indicating the applicability of the environmentally friendly particles for olefin-based drilling fluids.

## 1. Introduction

Drilling fluids are complex mixtures of solids and liquids dispersed or dissolved in water or oil phase, which play several fundamental roles during the drilling of gas and oil wells. These include (a) removal of cuttings generated by the drill bit from the borehole to the surface; (b) maintenance of the cuttings in suspension to avoid their sedimentation during a drilling stop; (c) cooling and lubricating the drill bit; (d) prevention of excessive fluid filtration through a permeable rock matrix by creating a filter cake sealing the rock pores, which can stabilize the wellbore and avoid its collapse. Each rock matrix requires the use of an appropriate drilling fluid formulation that suits its properties. Water-based drilling fluids (WBDFs) are preferred considering environmental and economic effects, whereas oil-based drilling fluids (OBDFs) are normally required under high-temperature and high-pressure (HTHP) conditions and in wells of water-sensitive clays/shales, and provide better lubricity and prevent corrosion of metal parts [1,2,3].

The advances in drilling operations under severe conditions have posed challenges for fluid formulations, as they must perform efficiently without causing environmental or health damage. Synthetic-based drilling fluids (SBDFs) are a class of high-performance oil-based drilling fluids with low environmental impact, since their continuous phase does not contain aromatics, and are composed of esters, ethers, linear alpha olefins (LAOs), isomerized olefins (IOs), or poly-alpha olefins (PAOs), which are more readily biodegradable and relatively nontoxic to most forms of life. However, chemical degradation and instability still occur when drilling through high-temperature and high-pressure formations [4,5,6].

In an attempt to overcome this limitation, various studies have recently reported the use of nanoparticles, such as SiO_2_, TiO_2_, Fe_2_O_3_, and carbon nanotubes, as additives for drilling fluid formulations. Because of their high surface-to-volume ratio, nanoparticles may provide superior fluid properties at low concentrations of additives, as well as better thermal stability. The benefits include optimal rheological and filtration characteristics, an increase in shale stability, and wellbore strengthening [3,7].

The ability of nanoparticles to seal small pores of shales and build an effective filter cake is higher than that of traditional larger particles, leading to enhanced fluid loss control, essential to maintaining formation stability and drilling productivity. However, most studies have used non-biodegradable and/or inorganic nanoparticles, which are harmful to health [7,8,9].

Cellulose nanocrystals (CNCs) are rod- or needle-like nanoparticles derived from the hydrolysis of the most abundant natural polymer, cellulose, which combine low cost, sustainability, biocompatibility, biodegradability, and surface functionalities, which make them excellent candidates for drilling operations [10]. However, CNCs are still little explored as additives for drilling fluids. Two recent papers by Li et al. [11,12] revealed that the use of CNCs on bentonite water-based drilling fluids improved the rheological and filtration properties of the fluids. However, no studies were found on the use of CNC-based particles in SBDFs.

Due to the intrinsic hydrophilicity of CNCs, surface chemical modification should be performed to provide the appropriate amphiphilicity needed to apply the CNCs in water-in-oil emulsions, such as SBDFs. Amphiphilicity can be introduced through poly(*N*-isopropylacrylamide) (PNIPAM) grafting from the CNC surface, promoting steric stabilization in an organic medium. PNIPAM is a thermosensitive and nontoxic polymer that shows an increased hydrophobicity upon heating (above 32 °C) and in the presence of salts, typical environments for SBDFs [13,14,15,16]. Recently, water-based drilling fluids with a bentonite surface modified with PNIPAM chains showed enhanced filtration control due to an improvement in the plugging of the mud cake by PNIPAM chains [17].

In this scenario, we aimed to prepare environmentally friendly CNC-*g*-PNIPAM copolymers and investigate their contribution to the properties of olefin-based drilling fluids.

## 2. Results and Discussion

### 2.1. Characterization of CNC and CNC-g-PNIPAM

**Figure 1:**
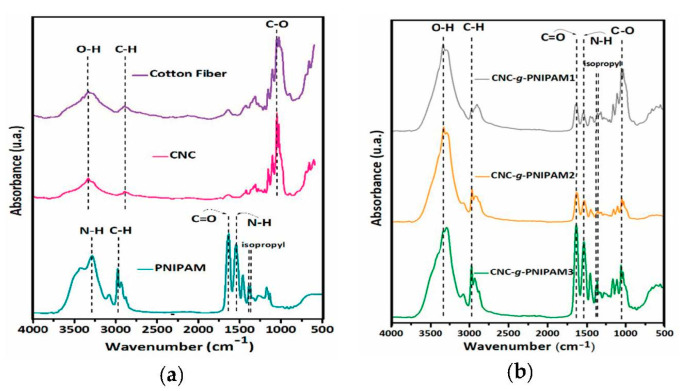
Infrared spectra of (**a**) cotton fibers, CNCs, and PNIPAM; (**b**) CNC-*g*-PNIPAM1, CNC-*g*-PNIPAM2, and CNC-*g*-PNIPAM3.

CNCs were grafted with poly(*N*-isopropylacrylamide) via free-radical initiation using the grafting from route. In this method, PNIPAM chains are formed from a CNC substrate by in situ polymerization. It is generally assumed that the radicals produced from the thermal decomposition of persulfate abstract the hydrogen from the hydroxyl groups of the polysaccharide to form alkoxy radicals on the substrate, which then attack the *N*-isopropylacrylamide (NIPAM) double bonds, leading to the polymerization. The first grafting indication of the modified CNC samples was verified by FTIR spectroscopy (Figure 1). All the spectra exhibited bands at about 3350 and 2900 cm^−1^, attributed to the O–H and C–H (saturated carbon) stretching vibrations, respectively, and a band at about 1050 cm^−1^ due to C–O stretching from the anhydroglucose units of cellulose [18,19,20].

The cellulose nanocrystals modified with PNIPAM presented, in addition to cellulose bands, the characteristic bands of PNIPAM at 1638 and 1541 cm^−1^, which denote, respectively, to C=O stretching (amide I) and the angular deformation of N–H (amide II), and peaks from the isopropyl group, at 1365 and 1385 cm^−1^, which indicate the functionalization of cellulose nanocrystals with PNIPAM chains [21,22].

**Figure 2:**
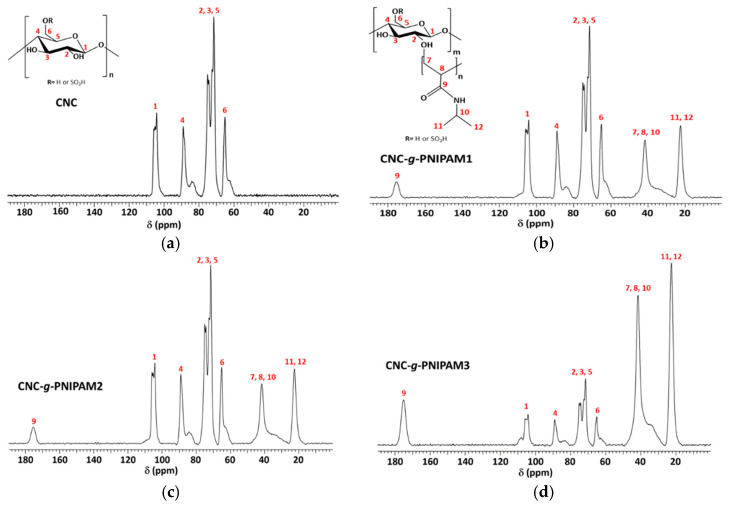
^13^C NMR spectra of (**a**) CNC; (**b**) CNC-*g*-PNIPAM1; (**c**) CNC-*g*-PNIPAM2; (**d**) CNC-*g*-PNIPAM3.

Further evidence of effective grafting was provided by solid-state NMR spectroscopy (Figure 2). All the spectra showed a peak at 65 ppm due to hydroxymethyl C6 carbons; a signal centered at 75 ppm, due to C2, C3, and C5 carbons; a peak at 89 ppm, due to C4 carbon; and a signal at 105 ppm, which corresponds to the C1 carbon from the cellulose repeat units. Upon grafting, new signals appeared from PNIPAM repeat units (Supplementary Figure S1 for PNIPAM spectrum): at 22 ppm, due to isopropyl groups (C11 and C12); at 41 ppm, ascribed to C7, C8, and C10 carbons; and a downfield peak at 175 ppm, attributed to a carbonyl group (C9). The absence of resonance of C=C carbons at 120–130 ppm indicated that the product is free from monomer impurities and that the purification was effective [23,24].

**Figure 3:**
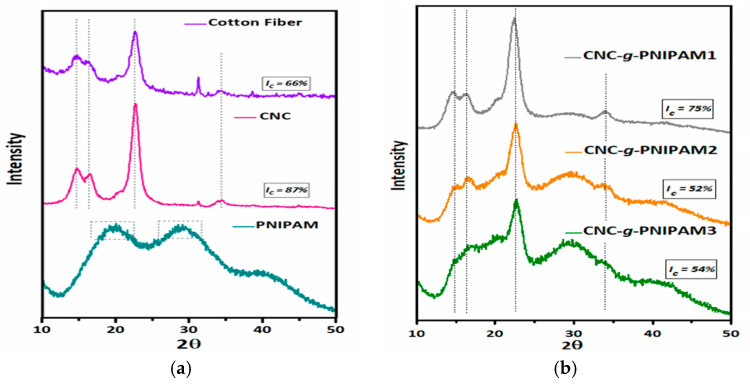
X-ray diffractograms of (**a**) cotton fibers (CF), cellulose nanocrystals (CNCs), and PNIPAM; (**b**) CNC-*g*-PNIPAM1, CNC-*g*-PNIPAM2, and CNC-*g*-PNIPAM3.

X-ray diffractograms of cotton fibers (CFs), CNCs, and CNC-*g*-PNIPAM copolymers are shown in Figure 3. The index of crystallinity (IC) increased from 66.15% for CFs to 87.73% for CNCs, implying the preference for acid attack on the amorphous regions of cellulose [16,18,19]. The CFs and CNCs exhibited peaks at 2θ of ~15°, 17°, 22.6°, and 34.5°, which showed that the crystalline structure of cellulose was not destroyed upon acid hydrolysis [19,25]. PNIPAM presented two broad diffraction signals, which indicated its amorphous structure, and the IC could not be determined. For the grafted nanocrystals, the IC decreased with increasing amount of NIPAM in the reaction medium. In the literature, similar behavior was observed for nanocrystalline cellulose modified with PNIPAM [20].

**Figure 4:**
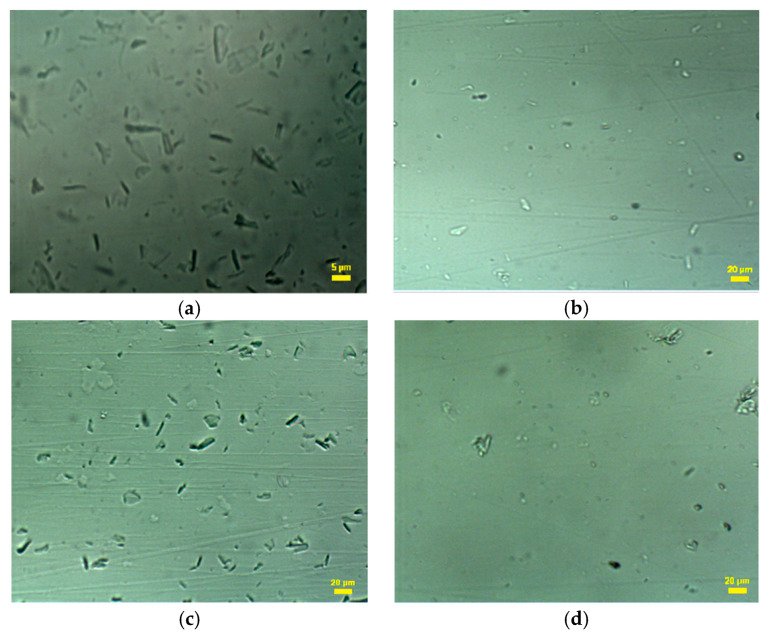
Confocal microscopy of the dispersion of (**a**) CNCs; (**b**) CNC-*g*-PNIPAM1; (**c**) CNC-*g*-PNIPAM2; (**d**) CNC-*g*-PNIPAM3.

The CNC and CNC-*g*-PNIPAM copolymer dispersions were visualized through confocal microscopy (Figure 4). The cellulose nanocrystals exhibited needle-shaped structures. The CNC-*g*-PNIPAM copolymers presented a higher degree of individualization among particles, probably because of the presence of surface polymer chains, generating entropic repulsion forces between the nanocrystals [26].

The surface properties of unmodified and functionalized CNC particles in aqueous dispersions were investigated by measuring the zeta potential in distilled water (Table 1). For the unmodified CNCs, the highly negative zeta potential was attributed to sulphate groups resulting from the sulfuric acid hydrolysis procedure. The grafted polymer brushes on the CNC surfaces masked the negative surface potential around the particles. Then, zeta potential values for the PNIPAM-grafted CNCs were lower (in modulus) than unmodified CNCs and decreased with increasing amounts of NIPAM added to the reactor feed. The values indicated that, after modification, CNC particles were no longer electrostatically but sterically stabilized [26,27].

The hydrodynamic diameters of the particles obtained from dynamic light scattering (DLS) measurements are also presented in Table 1 and in the Supplementary Materials (Figures S2 and S3). The small increase in the mean size of the filtered particles from CNCs to modified CNCs agrees with the change from bare to polymer-decorated nanocrystals [23]. In addition, to estimate the mean size of the particles after dispersion in the drilling fluid, the CNC-*g*-PNIPAM copolymers were added to distilled water and stirred at 20,000 rpm (the same as used for fluid preparation) and analyzed without a filtration step. The values were compared to the size of the particles dispersed by gentler magnetic stirring at 250 rpm (also without filtration). Although larger than the filtered particles, the mean size of the copolymers was much smaller when they were subjected to a high stirring speed, indicating disaggregation of the particles.

**Figure 5:**
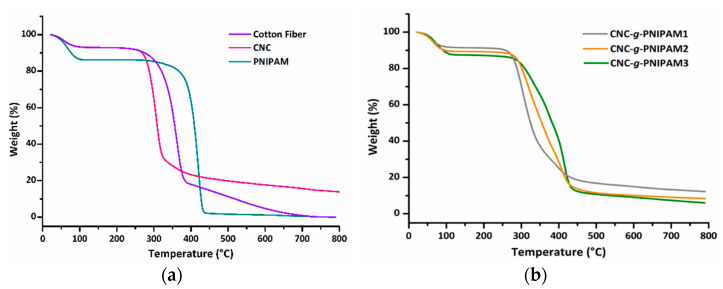
Thermogravimetric curves of (**a**) cotton fibers (CFs), cellulose nanocrystals (CNCs), and PNIPAM; (**b**) CNC-*g*-PNIPAM1, CNC-*g*-PNIPAM2, and CNC-*g*-PNIPAM3.

The thermal behavior of the solid state was studied by thermogravimetric analysis (TGA), and the results are presented in Figure 5. All the samples exhibited a mass loss below 100 °C, which was ascribed to moisture. The cotton fibers displayed a thermal degradation process in the 308–410 °C temperature range, related to cellulose degradation, which includes depolymerization, dehydration, and decomposition of glycosidic units. The CNCs exhibited a lower initial temperature of degradation (262 °C), attributed to the presence of sulfate groups, which reacted with the water released, leading to the production of sulfuric acid and, therefore, catalysis the degradation process [14,28].

Upon grafting, the CNCs increased their initial temperature of degradation to 273, 286, and 308 °C for CNC-*g*-PNIPAM1, CNC-*g*-PNIPAM2, and CNC-*g*-PNIPAM3, respectively. This behavior is attributed to the presence of PNIPAM chains, which start the degradation process 100 °C higher than pure CNCs and lead to surface-functionalized CNCs with improved thermal stability.

### 2.2. Characterization of the Olefin-Based Drilling Fluids

**Figure 6:**
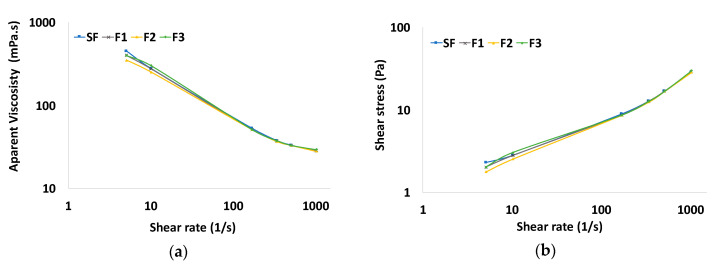
(**a**) Viscosity curves and (**b**) flow curves of the SF, F1, F2, and F3 drilling fluids.

Figure 6a shows the viscosity curves of olefin-based drilling fluids. All fluids presented shear-thinning behavior, with low viscosity at high shear rates, leading to good pumpability, and high viscosity at low shear rates, avoiding cutting accumulation in the borehole [2]. The viscosity values of F1, F2, and F3 were all similar to those of the standard fluid, indicating that modified nanocrystals do not disturb the rheological behavior of the fluid.

From the Bingham model plot of flow curves in Figure 6b, the plastic viscosity and yield point were determined and are presented in Table 1. These parameters also show that modified nanocrystals do not interfere with rheology. Li et al. [11] reported that the increase in the viscosity of water-based fluids with the addition of CNCs was a result of the creation of core–shell structures between bentonite (core) and CNCs (shell) through ionic surface interactions among bentonite layers, CNCs, and immobilized water molecules. Here, the lack of organoclay particles might have contributed to the rheological stability. High clay content can cause serious differential pressure sticking, well damage, and stuck pipe problems, especially under HTHP conditions. For this reason, high amounts of clay should be avoided and organoclay-free fluid formulations, such as the one used in this study, have been developed [29]. Besides, the PNIPAM grafts may have masked the sulfate groups and inhibited strong ionic interactions with other components of the fluid.

The electrical stability of a water-in-oil drilling fluid is essential to ensure the viability of drilling operations. The minimal electrical stability value recommended by the American Petroleum Institute (API) for non-aqueous drilling fluids is 200 V [30]. All the fluids showed elevated electrical stability values (Table 2). Probably, the amount of modified cellulose nanocrystals was not enough to result in a substantial difference in the electrical stability [31].

**Figure 7:**
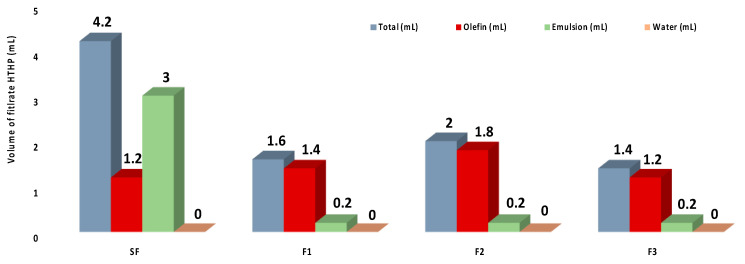
Volume of filtrate of the olefin-based drilling fluids after HTHP filtration.

The invasion of the liquid phase of the fluid (filtrate) into the rock formation must be controlled to avoid damage to oil- and gas-producing zones. During drilling, a filter cake is formed on the face by the components of the fluid, which can control fluids loss. Figure 7 shows the volume of the filtrate of the olefin-based drilling fluids after HTHP filtration. All the fluids produced with a small amount of CNC-*g*-PNIPAM presented much lower filtration loss than the standard fluid. No significant difference between F1, F2, and F3 filtrate loss was measured, and a minimal emulsion (0.2 mL) was found on the filtrate. It is assumed that the presence of modified cellulose nanocrystals can lead to a better closing of fluid penetration channels, leading to a thinner, impermeable filter cake and, subsequently, an improvement in the filtration properties [32,33,34,35].

**Figure 8:**
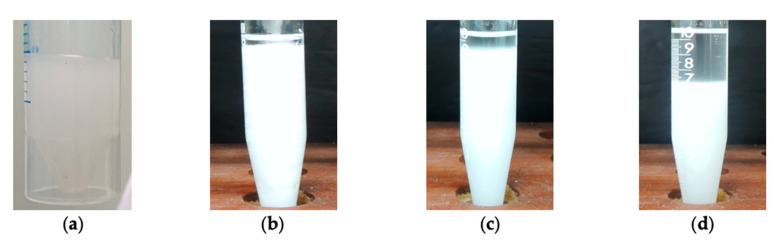
CNC-*g*-PNIPAM2 dispersed at (**a**) olefin; (**b**) 20/80 water/olefin; (**c**) 20/80 brine/olefin; (**d**) 40/60 brine/olefin.

To obtain better insight into the filtration behavior of the drilling fluids, water-in-oil dispersions (water/olefin = 20/80 and brine/olefin = 40/60 and 20/80) containing CNC-*g*-PNIPAM2 were prepared in Ultra Turrax^®^. The visual aspect of the systems is displayed at Figure 8. The CNC-*g*-PNIPAM2 formed an opaque dispersion immediately after stirring in olefin (Figure 8a), typical of certain affinity to the nonpolar medium, due to the presence of the -CH_2_-CHR- main chains from PNIPAM brushes. After adding distilled water, an outstanding gelled structure was formed (Figure 8b), suggesting polymer–water hydrogen bonding due to the amphiphilic character of PNIPAM. The literature states that filtration of a polymer-based fluid through a porous medium can be considerably reduced by increasing the elastic properties of the polymer [36]. The insertion of flexible PNIPAM chain grafts in the rigid structure of cellulose nanocrystals may produce the extensional viscosity needed to support the shear imposed during the filtration process at high pressure. In addition, a thin and elastic film (filter cake) of lower permeability also formed.

When brine (NaCl at saturation) was added (Figure 8c), a small amount of the olefin phase separated from the emulsion, similar to that observed for the water/olefin suspension. Phase separation increased when the brine/olefin ratio changed from 20/80 to 40/60 (the same applied to the fluids; Figure 8d). As isopropyl groups of PNIPAM contracted in the presence of NaCl, some olefin escaped to the filtrate. However, the amount of oil on the filtrate was low and would not compromise the rock formation stability. Further investigation is needed to improve the understanding of gelled polymer-decorated cellulose nanocrystal systems and the parameters that can control their performance on drilling fluids.

## 3. Materials and Methods

### 3.1. Materials

Cotton fibers (CFs) were purchased from York company (Brazil) and used as the cellulose source without any further purification. Sulfuric acid (hydrolysis agent) was purchased from Synth^®^ (Brazil). *N*-isopropylacrylamide (NIPAM, monomer) was supplied by Sigma Aldrich (Brazil), and potassium persulphate (K_2_S_2_O_8_, free-radical initiator) was purchased from Vetec Química Fina (Brazil).

Olefin (8-hexadecene), donated by PETORBRAS (Brazil), was used as the continuous phase of the synthetic-based drilling fluids. The following commercial additives were used to prepare the drilling fluids: BDF^−TM^ 997 (fatty acid polyamide, emulsifier), BDF^−TM^ 998 (fatty acid polyamide, emulsifier), lime (Cao, pH enhancer), ADAPTA^®^ L (polymer suspension, filtration control agent for organoclay-free oil-based drilling fluids), TAU-MOD^®^ (elongated inorganic particles, viscosifier for organoclay-free oil-based drilling fluids) [37], calcite (CaCO_3_, bridging agent), barite (Ba_2_SO_4_, weighting material), and sodium chloride (NaCl, for brine preparation). The additives were provided by PETROBRAS.

### 3.2. Preparation of Cellulose Nanocrystals (CNCs)

To prepare the CNCs, the cotton fibers were hydrolyzed based on the method described in the literature [38,39]. Briefly, 1 g cotton fibers was added to 30 mL of 65% (*w*/*w*) aqueous sulfuric acid solution. The reaction was left to proceed for 1 h at 55 °C under mechanical stirring. After this period, the reaction was interrupted by the addition of 30 mL cold distilled water. The material was centrifuged 4 times for 15 min at 9000 rpm. The suspensions of the nanocrystals were then dialyzed against distilled water until neutrality and the product was freeze-dried. The mass yield was 88%.

### 3.3. Surface Functionalization of Cellulose Nanocrystals (CNC-g-PNIPAM)

PNIPAM chains were grafted from cellulose nanocrystals (CNCs) by free-radical initiation, following literature methodology [20], but with some modifications. In this case, *N*-isopropylacrylamide and CNCs were added separately to distilled water under magnetic stirring for 30 min and 24 h, respectively. The resulting systems were mixed under mechanical stirring to produce a uniform suspension. Then, the suspension was heated to 60 °C, followed by the addition of K_2_S_2_O_8_ ([monomer]/[initiator] = 26/1), under N_2_(g) atmosphere. The reaction was left to proceed for 3 h. The materials were purified through dialysis against water and recovered by freeze-drying. The total concentration of NIPAM + CNC was 0.8% (*w*/*v*), according to the conditions presented in Table 3.

### 3.4. Infrared Spectroscopy

Infrared spectroscopy was performed on an IRAffinity-1 FT-IR spectrophotometer from Shimadzu. The samples were analyzed in KBr pellets scanned from 400 to 4000 cm^−1^.

### 3.5. NMR Spectroscopy

^13^C NMR measurements were performed on a Bruker Avance III HD 600 MHz spectrometer. The samples were analyzed in the solid state using a combination of cross-polarization with magic angle spinning (CP/MAS) with a spinning speed of 10 kHz, acquisition time of 30 ms, and contact time of 2 ms. The spectra were processed with the ACD/NMR Processor Academic Edition program.

### 3.6. X-ray Diffraction (XRD)

X-ray diffraction measurements were performed on a Bruker D2 Phaser diffractometer, operating with a CuKα radiation (λ = 1.54 Å) Ni filter at 30 kV and 10 mA, in the 2ϴ range of 10–50°, with a step size of 0.02°. The index of crystallinity (I_C_) was calculated using Equation (1), where I_1_ is the intensity of minimum diffraction, related to the amorphous region, and I_2_ is the intensity of maximum diffraction, related to the crystalline region [40].

I_C_ (%) = [1 − (I_1_/I_2_)] × 100 (1)

### 3.7. Zeta Potential

The zeta potential values were determined on a Stabino^®^ II, from Colloid Metrix. The samples were dispersed at a concentration of 0.1% (*w*/*v*) in distilled water, and measurements were performed in triplicate.

### 3.8. Dynamic Light Scattering (DLS)

Dynamic light scattering measurements were performed on a NANO-Flex^®^ II 180° DLS System, from Colloid Metrix, at 25 °C. The samples were dispersed in distilled water under magnetic stirring (250 rpm), overnight, at a concentration of 0.1% (*w*/*v*), and analyzed before and after filtration with 0.45 µm pore size cellulose acetate Millipore membranes.

In addition, to estimate the mean hydrodynamic diameter of the particles after dispersion in the drilling fluid, the polymers were added to distilled water (0.71 g/L, the same as used for drilling fluid) and stirred in an Ultra Turrax^®^ T25 digital, from IKA^®^, for 120 s at 20,000 rpm (the same as used for fluid preparation) and analyzed without a filtration step.

### 3.9. Confocal Microscopy

CNC and CNC-*g*-NIPAM suspensions were dispersed in Milli-Q^®^ water under magnetic stirring (250 rpm) overnight at a concentration of 0.014%. The samples were deposited in a glass plate and visualized on a confocal Raman microscope (LabRAM HR Evolution, HORIBA Scientific).

### 3.10. Thermogravimetry

Thermogravimetric analysis (TGA) was carried out on an SDT Q600 thermal analyzer from TA Instruments, at a temperature ranging from 30 to 800 °C, with a heating rate of 10 °C/min, and under nitrogen flow of 50 mL/min.

### 3.11. Preparation of the Drilling Fluids

The drilling fluids were prepared in a Hamilton Beach blender from Fann, with the compositions shown in Table 4. After blending, the formulations were subjected to dynamic aging for 16 h at 93 °C (200 °F) in a Fann roller oven to simulate the wellbore conditions. The samples were then studied by the recommended tests of the API RP 13B-2 standard for water-in-oil drilling fluids [30].

### 3.12. Rheology

The rheological properties of the fluids were determined on a Fann viscometer, model 35-A, equipped with coaxial cylinders, at 49 °C (120 °F). The fluid was submitted to the following decreasing rotational velocities: 600, 300, 200, 100, 6, and 3 rpm, equivalent to shear rates of 1021.8, 510.9, 340.6, 170.3, 10.2, and 5.1 s^−1^, respectively. The corresponding values obtained from the dial of the equipment were employed to calculate the apparent viscosity, plastic viscosity, and yield point.

### 3.13. Electrical Stability

The electrical stability values of the fluids were determined in a Fann model 23-E electrical stability tester at 49 °C (120 °F).

### 3.14. High-Temperature and High-Pressure (HTHP) Filtration Tests

Static filtration tests were performed in a Fann HTHP filter press at 93 °C (200 °F) under a pressure differential of 500 psi for 30 min. The fluid was placed into the filter cell with no. 50 Whatman filter paper, which was then inserted into a preheated heating jacket. The system was sealed and pressurized with N_2_(g). The filtrate was collected in a graduated test tube.

### 3.15. Preparation of Water in Oil Dispersions

Surface-functionalized nanoparticles were added to olefin and stirred in an Ultra Turrax^®^ T25 digital, from IKA^®^, for 120 s at 9000 rpm. Then, water or brine was slowly added to the system while stirring for 120 s at 9000 rpm. The total polymer concentration was 1% (*w*/*v*). The water/olefin ratio was 20/80, and the brine (35 wt% NaCl)/olefin ratios were 40/60 and 20/80. The final aspect of the systems was registered by a Coolpix P510 camera, from Nikon.

## 4. Conclusions

Cellulose nanocrystals were produced from acid hydrolysis of cotton fibers. The resulting CNCs were decorated with PNIPAM chains via free-radical initiation. FTIR and NMR spectra confirmed successful grafting. The absence of resonance of C=C carbons at 120–130 ppm indicated that the purification procedure was effective. The index of crystallinity increased from 66.15% for cotton fibers to 87.73% for CNCs, implying acid attack on the amorphous regions of cellulose. For the grafted nanocrystals, the I_C_ decreased with an increase in the amount of NIPAM in the reaction medium. Thermogravimetry analysis showed that modified CNCs exhibit higher thermal stability than pure CNCs.

The high negative zeta potential in CNCs is due to the sulphate groups from the sulfuric acid hydrolysis. The modified CNCs presented lower zeta potential (in modulus), as the grafted chains on the CNC surfaces masked the negative surface potential around the particles. DLS showed a small increase in the size of the particles from CNCs to modified CNCs, which agrees with the change from bare to polymer-decorated nanocrystals.

The results of rotational rheology and emulsion stability were not significantly affected by the addition of modified CNCs. However, a distinguishably low volume of filtrate was obtained when a small amount of CNC-*g*-PNIPAM was added to the fluids. This behavior indicates the potential application of CNC-*g*-PNIPAM on olefin-based fluids under HTHP conditions. Further investigation is needed to improve the understanding of the effects of polymer-decorated cellulose nanocrystals on the properties of the fluids and will be the subject of a forthcoming paper.

## Figures and Tables

**Table 1:** Zeta potential (mV) and hydrodynamic diameter (nm) of unmodified and functionalized CNCs particles in distilled water.

Sample	Zeta Potential (mV)	Hydrodynamic Diameter (nm) before Filtration ^1^	Hydrodynamic Diameter (nm) after Filtration ^1^	Hydrodynamic Diameter (nm) without Filtration ^2^
CNC	−34.2	-	104.1	-
CNC-*g*-PNIPAM1	−24.0	631.0	129.4	259.8
CNC-*g*-PNIPAM2	−12.3	534.1	126.1	211.4
CNC-*g*-PNIPAM3	−7.6	323.5	128.3	185.2

^1^ Dispersed by magnetic stirring at 250 rpm. ^2^ Dispersed in an Ultra Turrax^®^ at 20,000 rpm.

**Table 2:** Plastic viscosity, yield point, and electrical stability of olefin-based drilling fluids.

Drilling Fluid	Plastic Viscosity ^1^ (mPa s)	Yield Point ^1^ (Pa)	Electrical Stability (Volts)
SF	25.2	3.3	466
F1	26.1	3.1	512
F2	25.8	2.9	462
F3	26.7	3.0	513

^1^ Obtained from the Bingham model plot of curves in Figure 5b.

**Table 3:** Amounts of reactants used to prepare the CNC-*g*-PNIPAM copolymers and PNIPAM.

Sample	CNC (g)	NIPAM (g)	K_2_S_2_O_8_ (g)	Mass Yield (%)
CNC-*g*-PNIPAM1	1.5	0.5	0.045	94
CNC-*g*-PNIPAM2	1.0	1.0	0.090	94
CNC-*g*-PNIPAM3	0.5	1.5	0.135	95
PNIPAM	0	2	0.180	88

**Table 4:** Compositions of the drilling fluids.

Order of Addition	Constituents	Stirring Time (min)	Standard Fluid (SF)	F1 ^1^	F2 ^2^	F3 ^3^	Units
1	Olefin	1	186.8	186.8	186.8	186.8	mL
2	BDF^−TM^ 997	3	7	7	7	7	g
3	BDF^−TM^ 998	3	3	3	3	3	g
4	Lime	5	5	5	5	5	g
5	ADAPTA^®^ L	5	2	2	2	2	g
6	Tau-MOD^®^	5	2	2	2	2	g
7	Brine (35 wt% NaCl)	10	127.3	127.3	127.3	127.3	mL
8	Calcite	5	10	10	10	10	g
9	CNC-*g*-NIPAM	5	-	0.25	0.25	0.25	g
10	Barite	10	72.6	72.6	72.6	72.6	g

The fluids used the following additives: ^1^ CNC-*g*-PNIPAM1; ^2^ CNC-*g*-PNIPAM2; ^3^ CNC-*g*-PNIPAM3.

## Data Availability

The data presented in this study are available in request from the corresponding author.

## Supplementary Materials

The following are available online at https://www.mdpi.com/1422-0067/22/1/352/s1, Figure S1: ^13^C NMR spectrum of PNIPAM, Figure S2: Hydrodynamic radius of the CNC-*g*-PNIPAM1, CNC-*g*-PNIPAM2 and CNC-*g*-PNIPAM3 copolymers in water after filtration with 0.45 µm pore size cellulose acetate Millipore membranes, Figure S3: Hydrodynamic diameter of (a) CNC, (b) PNIPAM and (c) CNC-*g*-PNIPAM3 as a function of temperature, without filtration.
