# Supplementary material for: Polymer-Decorated Cellulose Nanocrystals as Environmentally Friendly Additives for Olefin-Based Drilling Fluids
Source: Int J Mol Sci. 2020 Dec 31;22(1):352. doi: 10.3390/ijms22010352 (PMC7795750; doi:10.3390/ijms22010352)
Supplement: Supplementary file 1 [file ijms-22-00352-s001.pdf]

International Journal of *Molecular Sciences*

MDPI

Supplementary Material

Polymer-decorated cellulose nanocrystals as environmentally friendly additives for olefin-based drilling fluids

José Aurélio Pinheiro[1], Nívia do Nascimento Marques[1], Marcos Antônio Villetti[2] and Rosangela de Carvalho Balaban[1]*

1 Laboratório de Pesquisa em Petróleo, Universidade Federal do Rio Grande do Norte, Natal-RN, 59078-970, Brazil; joseaureliopinheiro@gmail.com (J. A. P.); nivianmarques@hotmail.com (N. N. M.)
2 Universidade Federal de Santa Maria, Santa Maria-RS, 97105900, Brazil; mvilletti@hotmail.com
* Correspondence: rosangelabalaban@hotmail.com; Tel.: +55-84-3342-2323

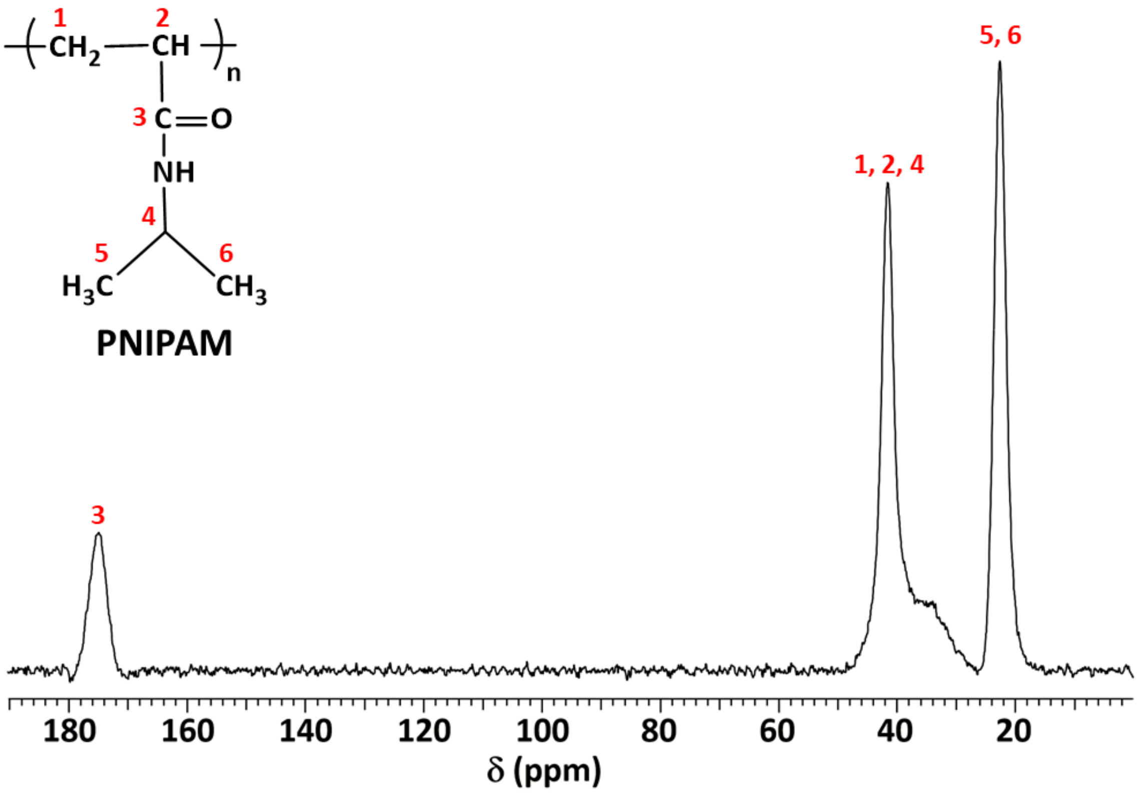

Figure S1. ^{13}C NMR spectrum of PNIPAM.

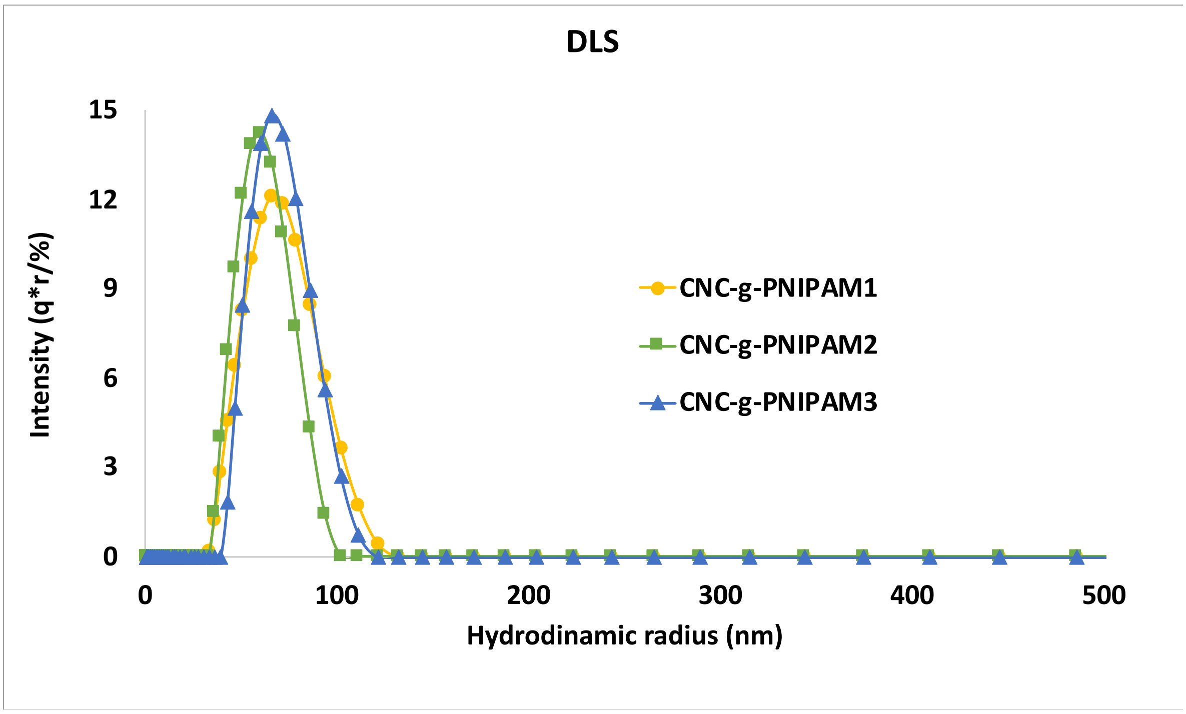

Figure S2. Hydrodynamic radius of the CNC-*g*-PNIPAM1, CNC-*g*-PNIPAM2 and CNC-*g*-PNIPAM3 copolymers in water after filtration with 0.45 μm pore size cellulose acetate Millipore membranes.

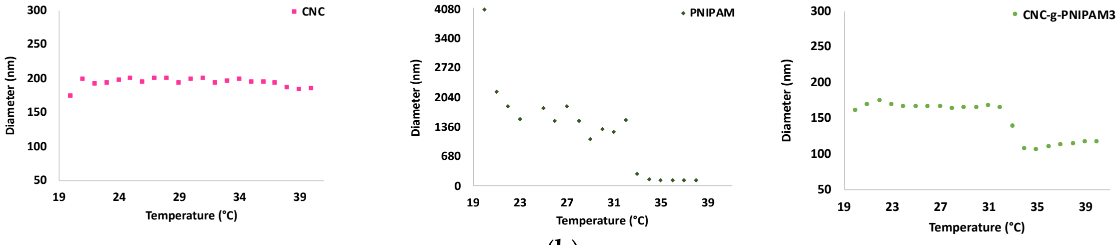

(**a**) (**b**) (c)

Figure S3. Hydrodynamic diameter of (**a**) CNC, (**b**) PNIPAM and (**c**) CNC-*g*-PNIPAM3 as a function of temperature, without filtration. As expected, CNC did not exhibit a thermosensitive behavior in water and the transmittance remained constant throughout the heating process. PNIPAM exhibited a large mean size, probably due to intermolecular associations, which abruptly decreases with heating, due to breakage of polymer-water and polymer-polymer intermolecular hydrogen bonds and increase of polymer-polymer intramolecular interactions when temperature reaches 32 °C. CNC-*g*-PNIPAM3 presented a much smaller size when compared to PNIPAM, but the thermosensitive behavior can still be observed by heating, as they exhibited a smaller size with the increase in temperature from 33 °C, due to the contraction of the PNIPAM chains decorating the cellulose nanocrystals.
